# Sensing Properties of Oxidized Nanostructured Silicon Surface on Vaporized Molecules

**DOI:** 10.3390/s19010119

**Published:** 2019-01-01

**Authors:** Nikola Baran, Hrvoje Gebavi, Lara Mikac, Davor Ristić, Marijan Gotić, Kamran Ali Syed, Mile Ivanda

**Affiliations:** Laboratory for Molecular Physics and Synthesis of New Materials, Ruđer Bošković Institute, Bijenička 54, 10000 Zagreb, Croatia; nikola.baran@irb.hr (N.B.); hrvoje.gebavi@irb.hr (H.G.); lmikac@irb.hr (L.M.); davor.ristic@irb.hr (D.R.); gotic@irb.hr (M.G.); kamran.syed@irb.hr (K.A.S.)

**Keywords:** porous, silicon, sensors, gas, ammonia, solvents, organic, oxidized

## Abstract

Porous silicon has been intensely studied for the past several decades and its applications were found in photovoltaics, biomedicine, and sensors. An important aspect for sensing devices is their long–term stability. One of the more prominent changes that occur with porous silicon as it is exposed to atmosphere is oxidation. In this work we study the influence of oxidation on the sensing properties of porous silicon. Porous silicon layers were prepared by electrochemical etching and oxidized in a tube furnace. We observed that electrical resistance of oxidized samples rises in response to the increasing ambient concentration of organic vapours and ammonia gas. Furthermore, we note the sensitivity is dependent on the oxygen treatment of the porous layer. This indicates that porous silicon has a potential use in sensing of organic vapours and ammonia gas when covered with an oxide layer.

## 1. Introduction

The growing importance of environmental monitoring, advances in process control in food and chemical industries, and stricter safety practices regarding new energy sources make gas sensors a key element for emerging technological solutions. The numerous applications of gas sensors include the need for both stationary and mobile sensing devices. While stationary devices (e.g., installed in a building) have somewhat looser requirements on size and power consumption, mobile sensing devices have to be relatively small and efficient to be portable for a reasonable amount of time.

Due in part to their large specific surface area, porous materials display properties useful in sensing applications. A variety of implementations are being explored, including porous gold as a biosensing platform ([1]), an electrochemical bisphenol A detector ([2]), and porous metal oxide semiconductors as gas sensing elements ([3]). In semiconductor sensors the gas comes in direct contact with the sensing element, causing a change in its electrical resistance. One of the most common semiconductor gas sensors is based on tin–dioxide (SnO_2_). Although (metal oxide) semiconductor–based gas sensors (e.g., TiO_2_, SnO_2_, ZnO) are already in use, they require moderate to high operating temperatures, ranging from ∼200–500 °C (see, e.g., [4,5,6] for review), rendering them unsuitable for low–power applications. Also, the price of sensing devices plays a role, leaning towards their cheaper production, and one easily implemented into existing industrial processes.

Despite the general lack of scientific and technical interest in porous silicon (PS hereafter) for the three decades after its serendipitous discovery in 1956, in the 1980s the interest is renewed, and many different applications of PS were found. This wide spectrum includes biomedical (e.g., [7]), photovoltaic (e.g., [8]), and sensor (e.g., [9]) applications. Also, various surface morphologies produced by electrochemical etching of PS have been studied ([10]). More specifically, PS makes an interesting basis for sensors due to a large specific area and compatibility with, now highly developed, silicon–based semiconductor production processes.

Efforts to produce room–temperature gas sensors are ongoing and include PS as a substrate with a goal of hydrogen (e.g., [11,12]), NO_2_ (e.g., [13,14,15,16]), and organic vapours (e.g, [17,18,19,20,21]) detection.

Observed responses in the above mentioned literature are electrical, although PS based sensors with optical response have also been studied (e.g, [22,23,24]). Even though oxidation of the porous layer is often regarded as an undesirable process (e.g., [25]), oxidized PS (OPS hereafter) has also been studied in the context of an optical gas sensor ([26]).

Recently, there is a rising interest in functionalization of PS with a goal of improving sensor selectivity. Examples include polypyrrole-coated porous silicon surface in CO_2_ detection ([27]), and TiO_2_ functionalized PS in specific detection of alcohols ([28]).

We find the literature lacking with respect to PS sensors functionalized with an oxide layer and OPS sensors with electrical response. Thus, in this work we study the resistive response of PS and OPS layers upon exposure to organic vapours and ammonia gas and study a possible novel way of PS functionalization.

## 2. Materials and Methods

In this section we describe the equipment used to fabricate OPS samples (Section 2.1), the Method for OPS sample preparation, their morphology and composition (Section 2.2). The experimental setup and equipment used to measure the response of the sample are described in Section 2.3, followed by the description of the measurement process in Section 2.4.

### 2.1. Etching Equipment

**Figure 1:**
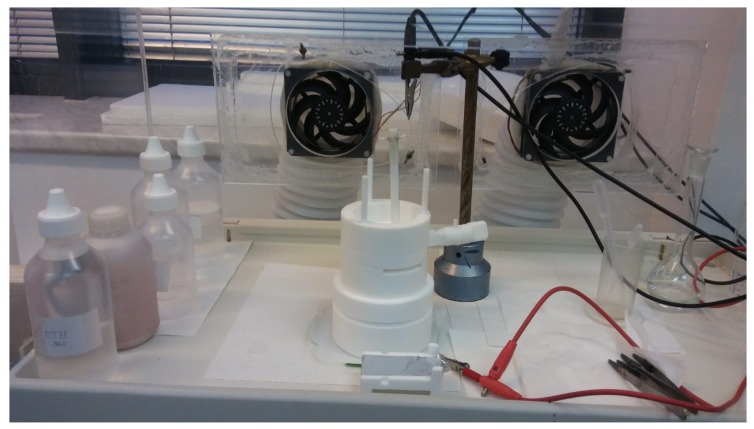
Polytetrafluoroethylene (PTFE) etching chamber. See Section 2.3 for details.

Samples were etched in an electrochemical process involving a dense platinum mesh cathode, and the piece of silicon wafer as the anode. The polytetrafluoroethylene (PTFE) etching chamber was custom–made, and consists of an electrolyte containment bowl (recommended electrolyte volume is 20 cm^3^) and a sample holder. The sample is exposed to the electrolyte through a hole at the bottom of the bowl, and makes electrical contact to a piece of thin aluminium foil on the underside. The electrolyte containment bowl has a groove to fasten the platinum electrode firmly in place, always at the same height above the silicon wafer piece. Also fitted to the bowl is a specially made PTFE valve for controlled removal of used electrolyte. The etching chamber is shown in Figure 1. As the constant current source we used a Keithley SourceMeter 2400 multi–purpose device. The device was programmed to output a desired current (28 mA) and connected to the platinum and aluminium electrodes of the etching chamber.

### 2.2. Sample Preparation

Samples were produced from p–type silicon wafers with resistivity of 0.005 Ωcm. The wafers were cut to ∼2 × 2 cm pieces and cleaned with isopropanol and acetone in hot ultrasonic bath for a total of 30 min. When dry, the pieces were put into a Diener Electronic ZEPTO plasma cleaner for 5 min. Cleaned pieces were then etched in 3:8 (*V*/*V*) concentrated (48%) hydrofluoric acid in ethanol solution. Etching was done in a custom–made PTFE chamber (described in Section 2.1), using an etching current density of 14 mA/cm^2^, for 5 min. After etching, to prevent the damage to the nanostructures due to surface tension, samples were washed in *n*–pentane. Some of the samples were placed into an oven, which was then evacuated to 14 Pa. The samples were then treated with a 12 SCCM flow of 99.999% pure oxygen for one hour, at a temperature of 180 °C and pressure of 48 Pa in a tube furnace. Meanwhile, other samples were left in the atmosphere.

**Figure 2:**
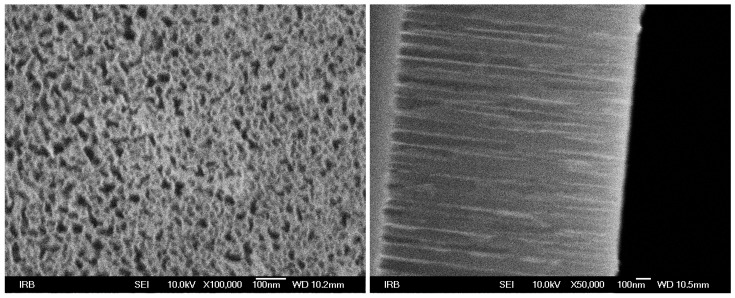
(**Left**) Scanning electron microscope (SEM) image of a sample after etching. (**Right**) Cross-sectional view of a sample showing the ∼1.6 μm thick porous layer on bulk silicon wafer. For details see Section 2.2.

After etching, the surface of the silicon wafer pieces visibly changed. We analysed it using a scanning electron microscope (SEM), showing pores which measure ∼23 nm in diameter, and 1.6 μm in depth, on average (see Figure 2). Energy Dispersive X–ray Spectroscopy (EDS) performed face–on with respect to the porous layer showed compositions of oxidized and non-oxidized samples as SiO_0.22_ and SiO_0.05_, respectively. EDS of the cross section of the oxidized sample has shown the outer half (rightmost in Figure 2) has twice the oxygen concentration of the inner half (leftmost in Figure 2). From ellipsometry measurements at 830 nm using Bruggeman’s approximation ([29]) we estimate a 52% porosity of the layer. Finally, to make a stable base for electrical probes (i.e., to prevent the probes from damaging the porous layer), we made two contact points on each sample. These points were produced by manually applying a thin layer of conductive silver paint using a fine-point brush, and were spaced ∼6 mm apart.

### 2.3. Measuring Equipment

**Figure 3:**
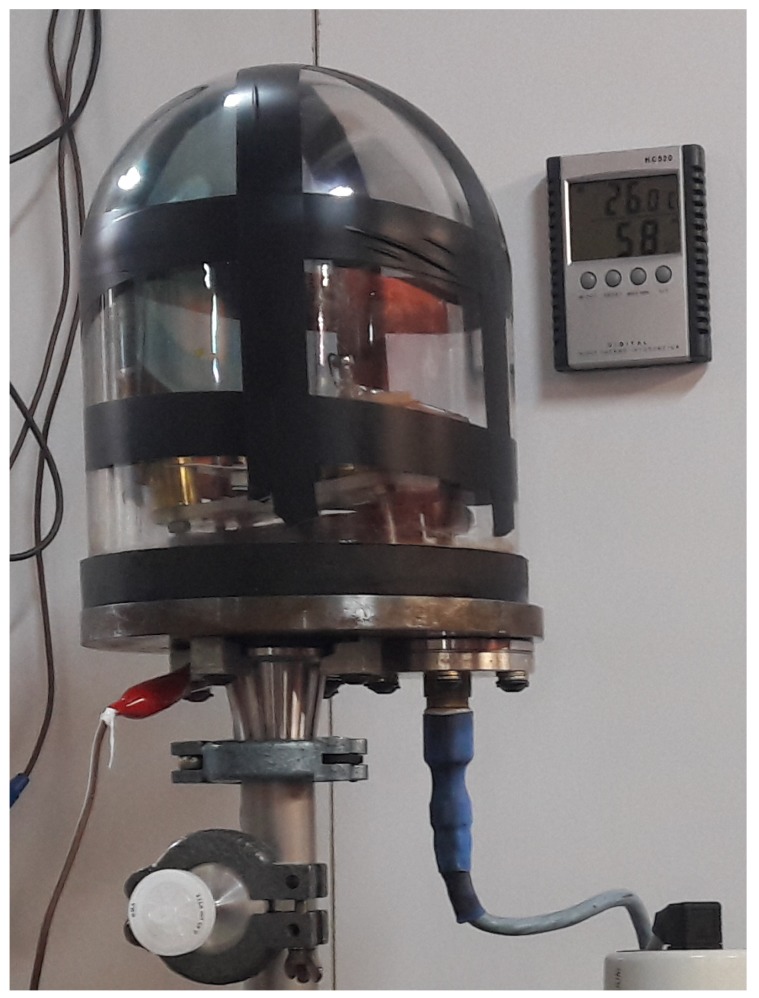
Chamber for measurements. The Pyrex glass jar, septum, and electrical contacts described in Section 2.3 are visible.

To assess the vapour concentration, we constructed a custom, hermetically sealed chamber of known volume. It consists of a Pyrex glass bell sealed to a steel bottom by a rubber gasket treated with Dow–Corning High Vacuum Grease. To be able to measure the electrical resistance, we made two electrically insulated and sealed contact ports in the bottom of the chamber. Also, a T–shaped fitting is screwed to the bottom, and connected to a PTFE septum and a vacuum pump. For a more efficient mixing of gases within the chamber, we placed a small electric fan powered by a brushless motor inside. The total volume of the chamber with the surrounding fittings is 4 dm^3^. The chamber is shown in Figure 3.

To measure resistance, we placed a sample holder equipped with thin electrical probes under the glass jar. The force exerted by probes onto the sample can be adjusted, which is important so as to avoid the change in electrical resistance due to collapse of the porous structures under the silver pads.

Probes were connected to the Keithley SourceMeter 2450, which measured and logged the DC ohmic (Ohmic resistance *R* is defined as the ratio between the measured voltage *V* and the constant sourced current *I* (R=V/I), and remains constant regardless of the sourced current.) electrical resistance *R* between the two contact points on the sample. To inject test gases and liquids, we used calibrated microliter syringes.

### 2.4. Measurements

We placed the sample into the holder and carefully lowered the probes onto the silver contacts. Upon closing the chamber we recorded the resistance in the atmosphere of air trapped within the chamber (ambient air). Using the hygrometer installed next to the chamber we measured the relative humidity of ∼50%. Then, the syringe was filled with a certain amount of liquid organic solvent at ambient temperature, and injected into the chamber through the septum. With each injection we recorded the injected volume of the solvent and ambient temperature. If the electrical resistance change was measurable (i.e., the change in resistance was larger than the noise), the next injection followed only after the resistance stabilized. We define the response of the sensor as a measurable change in resistance of the sample upon exposure to vapour or gas.

When there was no more response, we ended the measurement and ventilated the chamber. Then, after leaving the sample for approximately 16 h at atmospheric pressure and ambient temperature, with minimal exposure to the atmosphere surrounding the chamber, we restarted the measurements using another solvent. The experiments were performed three times.

To measure the response of the sample we used the following solvents: Ethanol (GramMol), methanol (Kemika), isopropanol (J. T. Baker), acetone (GramMol), n–pentane (Fisher Scientific), toluene (Macron), and chlorobenzene (Fisher Scientific). All solvents were of analytical purity. Additionally, we tested the response to 99.98% ammonia gas (Messer).

## 3. Results

In this section we present the results of electrical resistance measurements and their analysis.

On one hand, samples which were not treated with oxygen (see Section 2.2) gave no response, i.e., there was no measurable change in electrical resistance upon injecting the solvent into the chamber. On the other hand, samples which were oxidized responded with an increase in resistance each time a new volume of solvent was injected into the chamber. To gain insight into this effect, an oxidized sample was tested in detail.

**Figure 4:**
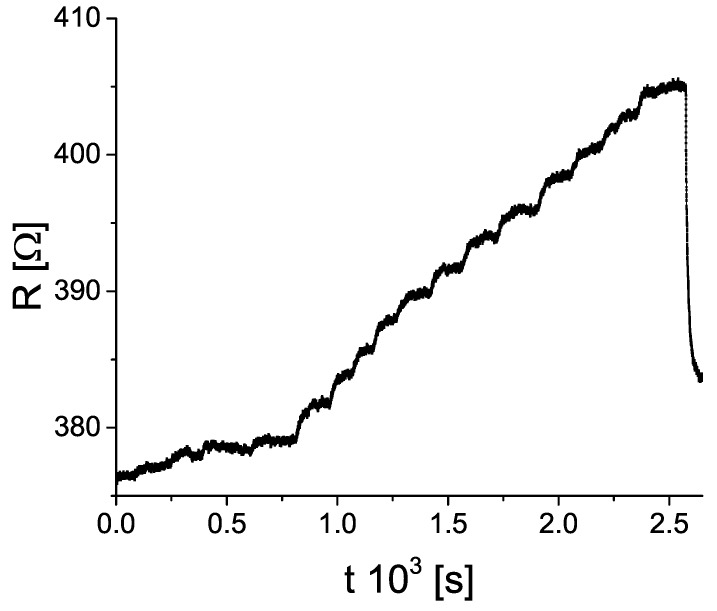
Resistance of the oxidized sample upon exposure to increasing concentration of methanol vapour. For details see Section 3.

A notable feature of the sample’s resistance is the rise immediately after the injection of the solvent (gas) into the chamber. To illustrate this, we show Figure 4. It is important to note this effect was not recreated upon injecting ambient air. After some time (in the range from 2 to 12 min, depending on the substance), the rise in resistance slowed down, and then stopped. The same behaviour was recorded for all of the tested substances (solvents and ammonia gas), however, with different rise times and step height. The response to injection of n–pentane was plagued by a high level of noise, and did not show the behaviour similar to other solvents (in fact, the resistance showed a hint of decrease with repeated n–pentane injections). For that reason, and to make analysis more clear, we decided to leave the measurements with n–pentane aside for the most of the following text.

For all of the tested compounds (except ethanol and ammonia gas (upon ventilating the chamber following the measurements with ethanol and ammonia gas, noise rose drastically.)) the resistance dropped nearly to the initial value minutes after chamber ventilation. This might indicate a relationship between the organic solvent vapour (gas) concentration and the resistance of the sample.

To test the relationship between the injection of the solvent or gas and the observed rise in electrical resistance, we made further tests. Specifically, we sought to compare the resistance rise time and the evaporation time of solvents. For that purpose, we used another enclosed chamber of volume similar to the one of the electrical resistance measurement chamber, equipped with a Mettler–Toledo analytical scale. Onto that scale we injected the same volume of solvent for which the resistance rise time was calculated. Rise time was calculated as an average time required for the resistance to rise from 10% of the initial value to 90% of the final value, for each step.

**Figure 5:**
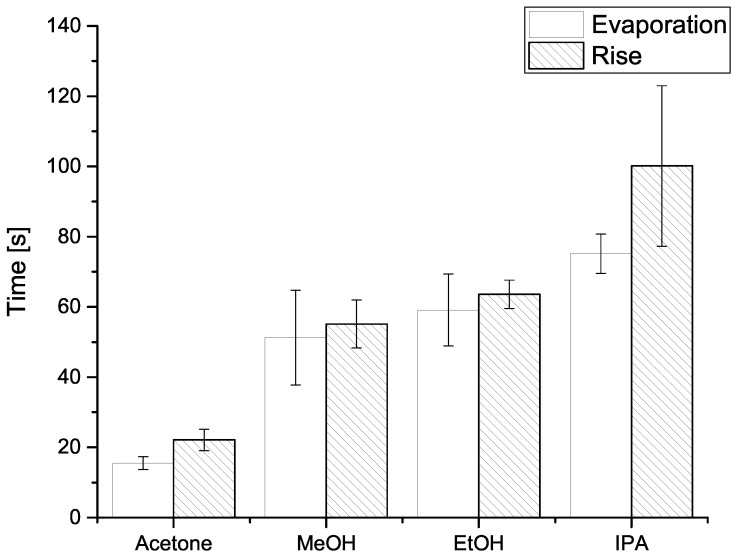
Histogram of mean evaporation time (blank) and resistance rise time (hatched) for (from left to right) acetone, methanol (MeOH), ethanol (EtOH), and isopropanol (IPA). On top of each column a range of one standard deviation is shown. Details are given in Section 4.

**Figure 6:**
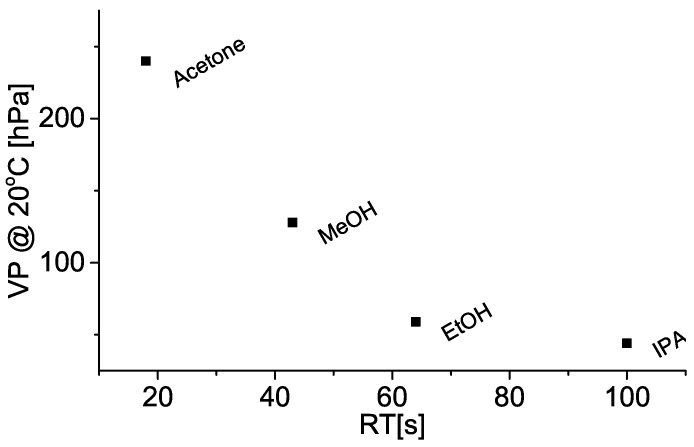
Dependence of solvent vapour pressure (VP) at 20 °C with respect to resistance rise time (RT) or (from left to right) acetone, methanol (MeOH), ethanol (EtOH), and isopropanol (IPA). Details are given in Section 4.

Comparison between resistance rise and evaporation times are shown in Figure 5, and indicates the two measured time values are comparable, i.e., within one standard deviation. Systematically shorter evaporation times (compared to resistance rise times) could be explained by the fact the analytical scale chamber was not hermetically sealed (unlike the resistance measurement chamber) and allowed some, however small, communication with the atmosphere, thus reducing the partial vapour pressure and facilitating evaporation. It is also important to note the solvents with higher vapour pressure both gave shorter signal rise times (Figure 6), and evaporated more quickly into the analytical scale chamber (Figure 5).

To calculate the vapour concentration (in ppm) from the known liquid solvent volume we used the relation given in [30]

(1) $$C=\frac{V_{L}\times D_{L}\times T_{L}}{M_{L}\times V_{A}}\times 8.2\times 10^{4}$$

where *C* is vapour concentration in parts–per–million (ppm), $V_{L}$, $D_{L}$ and $M_{L}$ are volume (in μL), density (in g·cm^−3^) and molecular mass of the solvent (in g·mol^−1^), *T* is the working (here: ambient) temperature, and $V_{A}$ volume of the chamber, i.e., gas (here: air) into which the liquid solvent evaporates.

Ammonia gas concentration was calculated using a simple ratio of the volume of the gas in the syringe and the volume of the chamber (in ppm). Table A1 contains data on injected volumes and corresponding organic vapour and ammonia gas concentrations.

**Figure 7:**
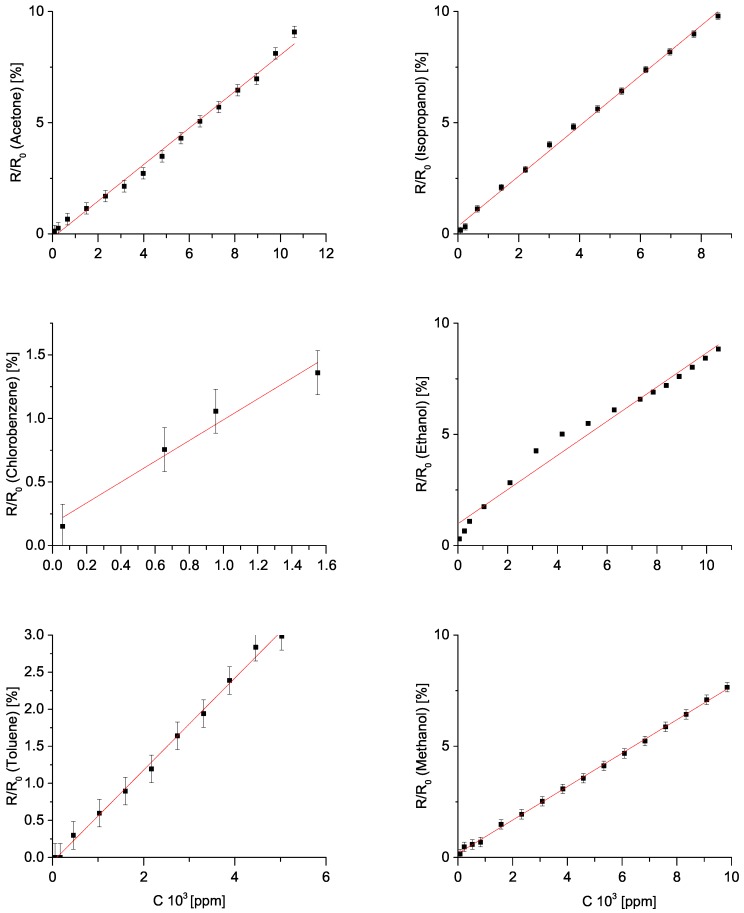
Dependence of electrical resistance of an oxidized sample on organic solvent vapour concentration (in ppm). From left to right and from top to bottom the plots represent the response to acetone, isopropanol, chlorobenzene, ethanol, toluene, and methanol, respectively. Error bars show five standard deviations. For details see Section 3.

**Figure 8:**
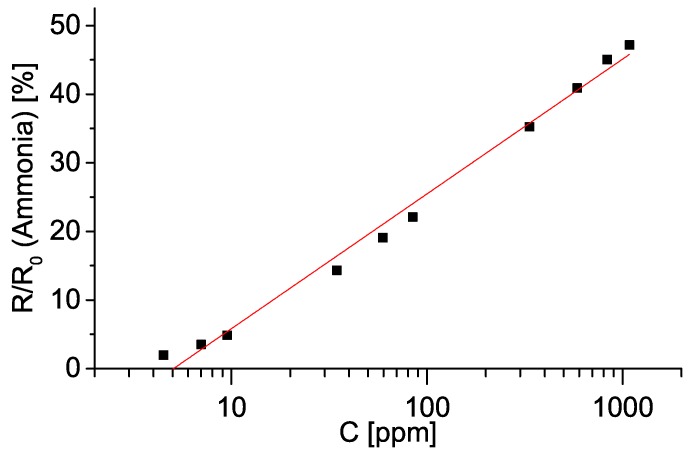
Dependence of electrical resistance of an oxidized sample on ammonia gas concentration (in ppm). Error bars showing five standard deviations are smaller than the size of the points. For details see Section 4.

First we analysed the relative change in resistance in dependence on vapour concentration. This is shown in Figure 7. Resistance values have been time–averaged over a plateau of each step, while vapour/gas concentrations were calculated as described earlier in this section. We tested a linear model of resistance as a function of concentration. $R^{2}$ values for such a model range from 0.97 for ethanol and chlorobenzene up to 0.99 for methanol and acetone. Thus, we conclude that a linear model describes the above mentioned relationship relatively well. Change in resistance upon exposure to ammonia gas showed logarithmic behaviour, as shown in Figure 8.

Then, we define the sensitivity as the slope of the fitted line. This value is a measure of relative change in resistance ($R/R_{0}$), where *R* is the resistance upon exposing the sample to organic vapour, and $R_{0}$ is the baseline resistance of the sample in ambient air) with respect to concentration in ppm. We express this sensitivity in percent per ppm. It ranges from 6 × 10^−4^%/ppm for toluene, up to 8 × 10^−4^%/ppm for acetone. Here we remind the reader that all of the measurements were conducted for n–pentane as well, however with high level of noise. If we were to calculate the sensitivity for n–pentane, it would be −3 × 10^−3^%/ppm.

## 4. Discussion

In this section we discuss possible physical mechanisms responsible for bonding of the tested molecules to the OPS surface, as well as those which might play a role in the change of electrical resistance.

### 4.1. Bonding Mechanism

Sensitivity values differed for various tested compounds (even after repeated measurements). This might indicate a connection between properties of a specific molecule and the change in resistance we observed. As we noted a quick and nearly complete recovery of the resistance after ventilating the organic vapours from the chamber, we would not expect chemical bonding of organic molecules to porous structure. For that reason, physisorption mechanisms (e.g., Van Der Waals or electrostatic bonding) are likely to be dominant in bonding of organic molecules to porous structures. Such bonding mechanisms have already been discussed ([17,31]).

Ammonia, however, produced somewhat different behaviour. Although the resistance of the sample did drop sharply after the chamber ventilation, the resistance quickly showed erratic and large oscillations. Moreover, after leaving the sample in ambient air for the usual ∼16 h, its sensitivity to organic vapours was negligible. This result is in agreement with the effect already noticed with ammonia gas being a “sticky” gas (e.g., [32]), suggesting chemisorption plays the dominant role in bonding of ammonia gas to our porous structures, showing permanent destructive behaviour (although after exposure to high ammonia concentrations). To gain insight into organic molecule bonding mechanisms, we studied the relationship between sensitivity and polarity of the molecules.

**Figure 9:**
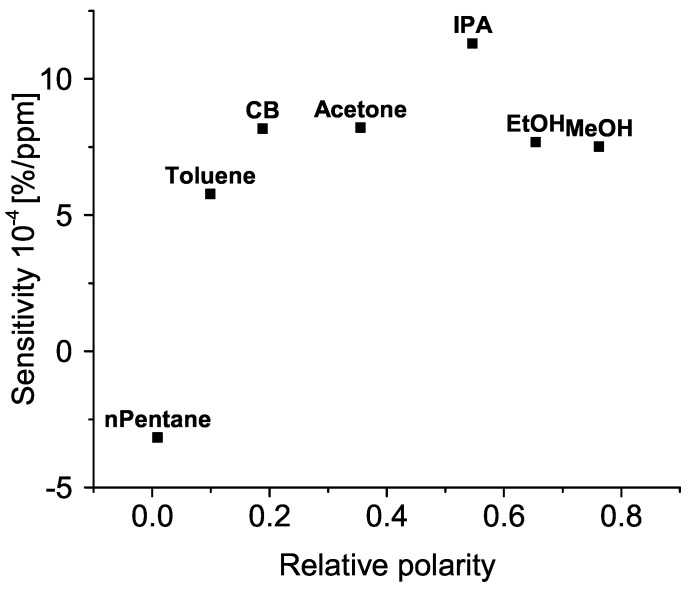
Sensitivity of an oxidized sample in dependence on relative polarity of organic molecules. CB, IPA, EtOH, and MeOH stand for chlorobenzene, isopropanol, ethanol, and methanol, respectively. See Section 4 for details.

This relationship is shown in Figure 9. A hint of positive correlation is visible between the sensitivity and relative polarity of tested molecules.

### 4.2. Sensing Mechanism

The physical mechanism responsible for the change in resistance is possibly a combination of dielectric properties of the adsorbed molecules, molecular kinematics and dipole effects. Such mechanism has been discussed by [17]. However, since there is a hint of positive correlation between the sensitivity and relative polarity of tested molecules we further discuss the dipole effect.

If the sensitivity and relative polarity of tested molecules are correlated, this might indicate that a mechanism related to the inherent electrical field of the molecule plays a dominant role in the change of resistance. This field would have narrowed the conductive channel between contacts, hindering the transport of charge carriers, and increasing the resistance. Such a picture is already present in the literature (e.g., [33]) where it is compared to a field–effect transistor with a “chemical” gate terminal. In that context, the higher the relative polarity of the bound molecule, the deeper its field could penetrate the charge transport layer. This is also in line with the results obtained by [34]. However, this model is highly dependent on the lowest point of relative polarity (n–pentane) for which the measurements are to be taken with a high degree of caution. Although the sensitivity to toluene is also lower than for other, more polar solvents, further measurements with low polarity solvents (e.g., hexane) are required to test this model and draw conclusions.

Additionally, the response might be improved with higher porosity and smaller pore diameter, due to the resulting larger surface area. While the pore diameter must remain larger than the kinetic radius of the tested molecules (e.g., 0.45 nm for ethanol), this requirement is easily exceeded. To optimize the sensor performance, this possible relation between sensitivity and pore geometry is to be studied in the future.

## 5. Conclusions

Oxygen treated porous silicon shows a potential in organic vapour and ammonia gas sensing. A rise in resistance as a response to increase in vapour/gas concentration is measurable from ∼50 ppm of organic vapours and ∼2 ppm of ammonia gas.

The response of the sample showed strong linear (logarithmic) behaviour for organic vapours (ammonia gas), throughout the measured range of concentrations. This puts this type of sensor closer to practical use, as it simplifies calibration. An additional advantage of oxygen treated porous silicon is its long–term stability in the atmosphere, avoiding possibly unpredictable behaviour due to slow oxidation.

Although the results are in line with the proposed chemical field–effect transistor model, bonding mechanisms, and the molecules’ effects on the charge carriers in the porous layer are not fully understood. Future experiments directed towards these particular points are required to shed light on these mechanisms and steer the development towards higher selectivity.

## Abbreviations

The following abbreviations are used in this manuscript:

CB	Chlorobenzene
EDS	Energy–dispersive X–ray spectroscopy
EtOH	Ethanol
IPA	Isopropanol
MeOH	Methanol
OPS	Oxidized Porous Silicon
PS	Porous Silicon
PTFE	Polytetrafluoroethylene

## Appendix A. Measurement Parameters

For completeness, in this appendix we give the injected volumes of organic solvents and ammonia gas, as well their concentrations as calculated from Equation (1).

**Table A1:** Table of injected volumes (in mm^3^) and the corresponding concentrations (in ppm) of liquid organic solvents and ammonia gas. See Section 2.4 for details.

Isopropanol		Acetone		Chlorobenzene		Ethanol		Toluene		Methanol		Ammonia	
V [mm^3^]	C [ppm]	V [mm^3^]	C [ppm]	V [mm^3^]	C [ppm]	V [mm^3^]	C [ppm]	V [mm^3^]	C [ppm]	V [mm^3^]	C [ppm]	V [mm^3^]	C [ppm]
79	1	1	83	60	1	0.5	52	57	1	7412	75	8	2
238	3	3	249	656	11	2.5	262	171	3	22,435	225	18	4.5
634	8	8	663	955	16	4.5	471	457	8	52,480	526	28	7
1426	18	18	1493	1552	26	10	1047	1028	18	82,526	826	38	9.5
2218	28	28	2322			20	2093	1600	28	157,641	1577	138	35
3009	38	38	3151			30	3140	2171	38	232,756	2329	238	60
3801	48	48	3980			40	4187	2742	48	307,871	3080	338	85
4593	58	58	4809			50	5233	3314	58	382,986	3831	1338	335
5385	68	68	5639			60	6280	3885	68	458,102	4582	2338	585
6177	78	78	6468			70	7327	4457	78	533,217	5333	3338	835
6969	88	88	7297			75	7850	5028	88	608,332	6084	4338	1085
7761	98	98	8126			80	8373			683,447	6835		
8553	108	108	8956			85	8897			758562	7587		
		118	9785			90	9420			833,677	8338		
		128	10,614			95	9943			908,792	9089		
						100	10,467			983,907	9840		
